# Seeking Positive Strengths in Buffering Athletes’ Life Stress–Burnout Relationship: The Moderating Roles of Athletic Mental Energy

**DOI:** 10.3389/fpsyg.2019.03007

**Published:** 2020-01-31

**Authors:** Shiow-Shya Chiou, Yawen Hsu, Yi-Hsiang Chiu, Chien-Chih Chou, Diane L. Gill, Frank J. Lu

**Affiliations:** ^1^Department of Physical Education, College of Sports and Recreation, National Taiwan Normal University, Taipei, Taiwan; ^2^Department of Physical Education, Health & Recreation, National Chiayi University, Taipei, Taiwan; ^3^Department of Physical Education, Chinese Culture University, Taipei, Taiwan; ^4^Graduate Institute of Sport Pedagogy, University of Taipei, Taipei, Taiwan; ^5^Department of Kinesiology, University of North Carolina at Greensboro, Greensboro, NC, United States; ^6^Graduate Institute of Sport Coaching Science, College of Education, Chinese Culture University, Taipei, Taiwan

**Keywords:** youth athletes, positive psychology, competitive sports, psychological well-being, optimal state of mind

## Abstract

In search of positive strengths that bolster athletes’ reaction to stress, the purpose of this study was to examine the moderating effects of athletic mental energy on the athletes’ life stress–burnout relationship. This study recruited two samples (Study 1 = 230; Study 2 = 159) and administered the College Student-Athlete’s Life Stress Scale (CSALSS; Lu et al., 2012), Athletic Mental Energy Scale (AMES; Lu et al., 2018), and Athlete Burnout Questionnaire (ABQ; Raedeke and Smith, 2001). Two separate hierarchical multiple regression analyses found that the emotional and cognitive components of athletic mental energy moderated the athletes’ life stress–burnout relationship across the two studies. Results provided the initial evidence that athletic mental energy can be positive strengths in buffering the stress–burnout relationship. Theoretical implications, limitations, practical applications, and future research directions are discussed.

## Introduction

Although sports professionals and physical educators suggest that engaging in competitive sports bring physical, social, and psychological benefits for the youth (Holt et al., 2011), it is reported that engaging in competitive sports is not totally beneficial. On their journey to athletic success, young athletes encounter many stressors that may endanger their physical and psychological well-being. These stressors include sport-specific stress (e.g., coach–athlete relationship, performance demands, training adaptation, and sports injury) and general life stress (e.g., interpersonal relationship, academic demands, romantic relationship, and family relationship) (Lu et al., 2012). In addition, in organizational sports, many environmental arrangements and operational procedures make competitive sports challenging and demanding. These organizational stressors include team selection, traveling, financial support, facilities adaptation, spectator pressure, rules and regulations, and competition format, which must be well-managed to avoid adverse consequences (Arnold et al., 2013; Fletcher et al., 2006). Furthermore, exacerbating athletes’ stress, it is reported that if young athletes want to be successful, they must start their training at a very young age, train all year-round, and sometimes with excessive training (Gustafsson et al., 2011).

Although stress is an inevitable part of life in general and competitive sports specifically, it is well-documented that excessive stress may lead to physical and mental illness/problems. In terms of physical problems, excessive stress may cause gastrointestinal ulcers (Marik et al., 2010), increase hyperglycemia (Bosarge and Kerby, 2013), elevate the possibility of asthma (Theoharides et al., 2012), and increase the risk of heart disease (Steptoe and Kivimaki, 2012). On the mental aspect, excessive stress is related to hopelessness and suicide ideation (Ibrahim et al., 2014), depression (Risch et al., 2009), eating disorders (DiBartolo and Shaffer, 2002), lower well-being (DiBartolo and Shaffer, 2002), decreased performance (Humphrey et al., 2000), and burnout (Gustafsson and Skoog, 2012; Lu et al., 2016).

Burnout is a serious condition that has received much attention by researchers because it could lead to athletes’ dropout and lower psychological well-being (Gustafsson et al., 2011). Athletic burnout is a complex psychophysical syndrome characterized by “… *feeling physically and psychologically exhausted from the demands of training and competing, perceive a reduced sense of accomplishment, and experience sport devaluation in which they engage*” (Raedeke and Smith, 2001, p. 283). Smith (1986) proposed a cognitive-affective model of athletic burnout in which burnout is a reaction to chronic stress. According to Smith (1986), athletes live in a harsh environment filled with conflicts and demands, such as meeting athletic and academic demands simultaneously or within a short time, team selection that requires high-performance records or high standards of physical fitness/skill tests, or dealing with interpersonal relationships within and outside sports. Under such conditions, Smith (1986) contends that athletes’ cognitive appraisals—evaluating the balance between challenges and resources, and potential consequences of not meeting the demands—lead to athletic burnout. Smith (1986) contends that these cognitive appraisals play a central role in the process. Specifically, when athletes perceive that demands surpass personal resources, and consequences will be severe, they have negative physical and psychological responses, such as anxiety, tension, insomnia, and illness. Finally, physiological and psychological responses lead to rigid and inappropriate behavior, decreased performance, and withdrawal from activity.

Past research adopting the Smith (1986) burnout model generally supported the link between stress and athletic burnout (Tabei et al., 2012; Chyi et al., 2017; Chang et al., 2018). It is imperative to understand the moderators/mediators underlying the stress–burnout relationship so that practitioners can use this knowledge to help athletes avoid burnout (Chang et al., 2017). Some researchers have investigated those mediators/moderators. For example, Gustafsson and Skoog (2012) sampled 217 young athletes to examine the mediating role of optimism in the stress and burnout relationship. They found perfectionism, perceived stress, and burnout all correlated, and perceived stress fully mediated the optimism–burnout relationship. Similarly, Chang et al. (2017) sampled 300 college student-athletes and measured life stress, negative thoughts, and burnout. They found life stress and negative thoughts positively correlated with burnout. Additionally, hierarchical regression analyses found that negative thoughts mediated the stress–burnout relationship.

Although examining factors that mediate the stress–burnout relationship is critical, it is even more important to understand factors that can change this stress–burnout relationship, which are moderators (Barron and Kenny, 1986, p.1174). In this line of research, researchers focus on athletes’ positive strengths/merits as a salient factor. For example, in a study that examined conjunctive effects of athletes’ resilience and social support in moderating the stress–burnout relationship, Lu et al. (2016) sampled 218 student-athletes and measured life stress, resilience, social support, and burnout. A series of one-, two-, and three-way interactions examined disconjunctive and conjunctive moderations. They found under high life stress condition, athletes’ resilience and coaches’ social support conjunctively moderated the stress–burnout relationship. Specifically, under high life stress conditions, athletes with high resilience and coaches’ high tangible social support were less susceptible to burnout than those with high resilience but low coaches’ tangible social support. Recently, Chang et al. (2018) used a two-wave, time-lagged survey to examine the moderating effects of psychological flexibility on the athletic identity–burnout relationship. They found that high athletic identity athletes with low psychological flexibility developed emotional exhaustion (one factor of burnout) over time, but high athletic identity with high psychological flexibility was negatively associated with emotional exhaustion over time.

Research on positive strengths/merits that moderate the athletes’ life stress–burnout relationship is insightful for the researchers in the sports domain. Specifically, as the world entered a new millennium, psychologists turned their focus away from treating mental illness to building strengths and virtues (Seligman, 2002). Many positive strengths, such as gratitude (e.g., Chang et al., 2018; Gabana et al., 2019), resilience (e.g., Lu et al., 2016; Hill et al., 2018), mindfulness training (Jouper and Gustafsson, 2013), intrinsic motivation (Cresswell and Eklund, 2006; Li et al., 2013), harmonious passion (Gustafsson et al., 2011), forgiveness (e.g., Watson et al., 2017; Akhtar and Barlow, 2018), and altruism (e.g., Feigin et al., 2018) have been examined by researchers in sports as well as in many domains. Therefore, sports researchers should continue to seek positive strengths/merits that may moderate the athletes’ stress–burnout relationship.

Recently, Lu et al. (2018) adopted the conceptual framework of mental energy proposed by the International Life Science Institute (ILSI; O’Connor and Burrowes, 2006, p.2) and developed a sport-specific construct termed “athletic mental energy,” which may be related to the athletes’ stress–burnout relationship. In mainstream psychology, mental energy is defined as “…*an individual’s ability to continue long hours of thinking, concentrating attention, and blocking distractions to achieve a given task* (Lykken, 2005).” Lykken (2005) contended that many great scholars, such as Archimedes, Galileo, Newton, and Einstein, create so many astonishing works because they have a strong mental energy. Lu et al. (2018) adopted the ILSI framework of mental energy and followed the guidelines suggested by the Standards for Educational Psychological Testing (American Educational Research Association, American Psychological Association, and National Council on Measurement in Education, 2014) to develop a sport-specific mental energy scale called Athletic Mental Energy Scale (AMES). Across six studies, Lu et al. (2018) found that the six-factor, 18-item AMES had appropriate validity and reliability. In particular, athletic mental energy negatively correlated to athletic burnout and life stress but positively with a positive state of mind (Lu et al., 2018; pp. 7–9).

Thus, we considered that athletic mental energy might play a moderating role between athletes’ life stress and burnout for several reasons. First, although Lu et al. (2018) found that athletic mental energy negatively correlates to athletic burnout and athletes’ life stress, the role of athletic mental energy in the stress–burnout relationship has never been fully examined. Second, athletic mental energy consists of positive components, which is in line with positive psychology. The emotional components of athletic mental energy, such as vigor and calm, are frequently reported in sports literature. For example, Brandt et al. (2016) found that champion rowers had higher vigor and lower depression and fatigue than those who are not champions. Similarly, Lane et al. (2017) conducted a large-scale Internet experiment (*n* = 73,568) and found that participants high in depression performed poorly in Internet games. In contrast, those high in vigor and low in depression performed better.

Furthermore, according to Lu et al. (2018), athletic mental energy also includes positive cognitions, such as confidence, motivation, and concentration. In the sports domain, these perceptions are associated with high performance. For example, Abdullah et al. (2016) recruited 26 Malaysian national soccer players to complete the Psychological Skills Inventory for Sports (PSIS; Mahoney et al., 1987) before the Malaysian super cup. Then, 10 experts judged their performance during the games. They found that participants’ motivation, self-confidence, anxiety control, preparation, and concentration predicted soccer performance. Similar studies have found that confidence, motivation, and concentration are the key factors associated with athletic success (e.g., Fransen et al., 2015; Lochbaum and Gottardy, 2015).

Based on the above literature, the purpose of this study was to examine the moderating effects of athletic mental energy on the stress–burnout relationship. We hypothesized that athletic mental energy moderates the stress–burnout relationship. We examined these relationships in two studies with two different samples.

## Materials and Methods

### Study 1

#### Purpose

The purpose of Study 1 was to examine the moderating effects of athletic mental energy on the athletes’ life stress–burnout relationship.

#### Methods

##### Participants

Participants were 230 college student-athletes (males = 164; females = 66) with a mean age of 19.92 years (SD = ±1.59) from 14 universities in Taiwan. At the time of the data collection, participants were all in their regular training seasons and had been participating in 25 varied individual and team sports, such as gymnastics, track and field, golf, weightlifting, basketball, volleyball, Tae-kwon-do, badminton, and baseball. The average participation years in competitive sports was 6.46 years (SD = ±4.02).

##### Measurements and Procedures

Prior to data collection, the researchers gained approval from a local institute ethical committee (TSMHIRB-2-R-030-2.1). Then, the first author contacted target teams’ coaches through e-mails and phone calls and briefly informed them of the purpose of the research, confidentiality, and anonymity for participation. After agreement, we made an appointment to collect data. A survey package included a demographic questionnaire and psychological scales [i.e., Athlete Burnout Questionnaire (ABQ), Athletic Mental Energy Scale (AMES), and College Student-Athletes’ Life Stress Scale (CASLSS)]. To prevent social desirability effects, we informed participants that this was a study to explore college students’ life experiences, that there were no right or wrong answers, and that all responses would be confidential. If they agreed, they signed the consent forms and were asked to answer the questions as truthfully as possible. The measures were as follows:

##### Demographic Questionnaire

The demographic questionnaire collected participants’ age, gender, types of sports, and years of athletic experiences.

##### ABQ

The ABQ (Raedeke and Smith, 2001) is a self-reported inventory that assesses athletes’ burnout experiences. The ABQ has three subscales including *reduced sense of athletic accomplishment*-sample question such as “I accomplish nothing from sports,” *perceived emotional and physical exhaustion*-sample question such as “I feel so tired from the training that I have trouble finding energy to do anything else,” and *devaluation of sports participation-*sample question such as “The effort I spend in sports would be better spent doing other things.” The ABQ used a six-point Likert scale from 1 (never) to 6 (always). Higher scores on the ABQ indicate that athletes are high in burnout. In this study, we used the total score of the ABQ for the main analysis, and its Cronbach’s α was 0.92.

##### CSALSS

The 24-item CSALSS (Lu et al., 2012) was used to assess situations that athletes encountered in their daily life and sports and considered as major stressors in their lives. The 24-item CSALSS has eight factors, including (a) sports injury, (b) performance demand, (c) coach relationships, (d) training adaptation, (e) interpersonal relationships, (f) romantic relationships, (g) family relationships, and (h) academic requirements. According to Lu et al. (2012), CSALSS can be categorized into two major components—sport-specific stressors (by adding factors a, b, c, d) and general life stressors (by adding factors e, f, g, h). Sample questions are: “I am annoyed with my coach’s bias against me” or “I am annoyed with my injuries.” Participants indicated the frequency of such experiences on a six-point Likert scale that ranged from 1 (never) to 6 (always). The Cronbach’s α of the two composite factors in this study were 0.85 and 0.86. We used the two composite scores of CSALSS for the main analysis.

##### AMES

The 18-item AMES (Lu et al., 2018) was used to assess an athlete’s perception of his/her existing energy state, which is characterized by the intensity in motivation, confidence, concentration, and mood. There are six factors in the 18-item AMES, each with three items, including (a) vigor (items 1, 2, and 15), (b) confidence (items 3, 9, and 13), (c) motivation (items 4, 8, and 16), (d) tireless (items 7, 11, and 12), (e) concentration (items 5, 6, and 10), and (f) calm (items 14, 17, and 18). When answering AMES, participants have to identify the feeling of each item on a six-point Likert scale that ranged from 1 (not at all) to 6 (completely so). The items and scoring for AMES are in Appendix A, which is provided by our correspondence author. The Cronbach’s α of the six factors of the AMES in this study ranged from 0.77 to 0.89, and the total score of AMES was 0.93. We used the six factors of AMES and the total score of AMES for the main analysis.

#### Statistical Analyses

We used Pearson correlation analysis to examine the relationships among the two composite scores of life stress, the six factors of mental energy, and the total burnout score. Furthermore, hierarchical regression analyses were used to examine the moderating effect of athletic mental energy on the stress–burnout relationship. To examine the main effects of life stress and athletic mental energy on burnout, two types of life stress (i.e., sport-specific and general life stress) and athletic mental energy (i.e., six factors of the AMES and the total score of the AMES) were entered in step 1. The interaction analysis was then entered in step 2 (i.e., sport-specific/general life stress × six components of athletic mental energy). The interaction scores were calculated by centering on reducing the collinearity between the independent variable and the interaction term (Aiken and West, 1991). The significance was set at *p* < 0.05. We used SPSS 18.0 statistical software for all analyses. Furthermore, to estimate the interaction effect, we followed Aiken and West’s (1991) equation to compute simple slopes when the interaction was significant.

#### Results

Table 1 indicates that all subscales exhibited good to excellent internal reliability (α = 0.77–0.93). Zero-order correlations show that all subscales of athletic mental energy were negatively correlated with burnout (*r* = −0.33∼−0.45, *p* < 0.01), and the two types of life stress were positively correlated with burnout (*r* = 0.37 and 0.39, *p* < 0.01). Table 2 indicates the moderating effects of athletic mental energy on the sport-specific stress–burnout relationship. Results show that five factors of athletic mental energy, all except concentration, moderated the sport-specific stress–burnout relationship as follows: vigor (β = −0.226, Δ*R*^2^ = 0.050, *p* < 0.01), confidence (β = −0.128, Δ*R*^2^ = 0.016, *p* < 0.05), motivation (β = −0.147, Δ*R*^2^ = 0.021, *p* < 0.05), tireless (β = −0.179, Δ*R*^2^ = 0.032, *p* < 0.01), calm (β = −0.139, Δ*R*^2^ = 0.019, *p* < 0.05), and total score of mental energy (β = −0.187, Δ*R*^2^ = 0.034, *p* < 0.01).

**TABLE 1:** Descriptive statistics and bivariate correlations for all study variables (Study 1).

	1	2	3	4	5	6	7	8	9	10
1. SS										
2. GLS	0.62**									
3. Vigor	−0.49**	−0.49**								
4. Confidence	−0.46**	−0.43**	0.64**							
5. Motivation	−0.33**	−0.34**	0.62**	0.64**						
6. Cconcentration	−0.45**	−0.33**	0.56**	0.60**	0.51**					
7. Tireless	−0.45**	−0.33**	0.52**	0.54**	0.41**	0.46**				
8. Calm	−0.37**	−0.37**	0.45**	0.60**	0.50**	0.63**	0.49**			
9. Mental energy	−0.54**	−0.48**	0.78**	0.84**	0.76**	0.80**	0.74**	0.79**		
10. Brunout	0.37**	0.39**	−0.36**	−0.33**	−0.40**	−0.33**	−0.39**	−0.31**	−0.45**	
Mean	2.45	2.16	4.33	4.01	4.67	4.03	3.49	3.99	4.09	2.72
SD	0.79	0.85	0.81	0.95	0.96	1.10	1.20	1.10	0.80	0.98
α	0.85	0.86	0.77	0.80	0.84	0.87	0.89	0.87	0.93	0.92

SS, sport-specific stressors; GLS, general life stressors. *p < 0.05, **p < 0.01.

**TABLE 2:** Summary results of the moderating effects (Study 1).

	**Step 1: direct effect**			**Step 2: interaction effects**			
	**β**	***t*-value**	***R*^2^**	**β**	***t*-value**	***R*^2^**	**Δ*R*^2^**
SS	0.250	3.61**	0.174	0.214	3.15**	0.224	0.050**
Vigor	–0.233	−3.37**		–0.231	−3.44**		
SS × Vigor				–0.226	−3.82**		
SS	0.269	3.95**	0.167	0.256	3.77**	0.183	0.016*
Confidence	–0.207	−3.04**		–0.209	−3.09**		
SS × Confidence				–0.128	−2.12*		
SS	0.261	4.21**	0.220	0.249	4.05**	0.241	0.021*
Motivation	–0.313	−5.04**		–0.291	−4.70**		
SS × Motivation				–0.147	−2.50**		
SS	0.270	3.99**	0.168	0.275	4.07**	0.179	0.011
Concentration	–0.209	−3.09**		–0.189	−2.76**		
SS × Concentration				–0.105	–1.71		
SS	0.237	3.57**	0.199	0.224	3.43**	0.230	0.032**
Tireless	–0.286	−4.31**		–0.292	−4.48**		
SS × Tireless				–0.179	−3.05**		
SS	0.289	4.44**	0.169	0.280	4.34**	0.188	0.019*
Calm	–0.204	−3.14**		–0.204	−3.16*		
SS × Compose				–0.139	−2.31**		
SS	0.172	2.48*	0.224	0.160	2.35*	0.258	0.034**
Mental energy	–0.357	−5.14**		–0.341	−5.00**		
SS × Mental energy				–0.187	−3.24**		

SS, sport-specific stressors; GLS, general life stressors. *p < 0.05, **p < 0.01.

**FIGURE 1:**
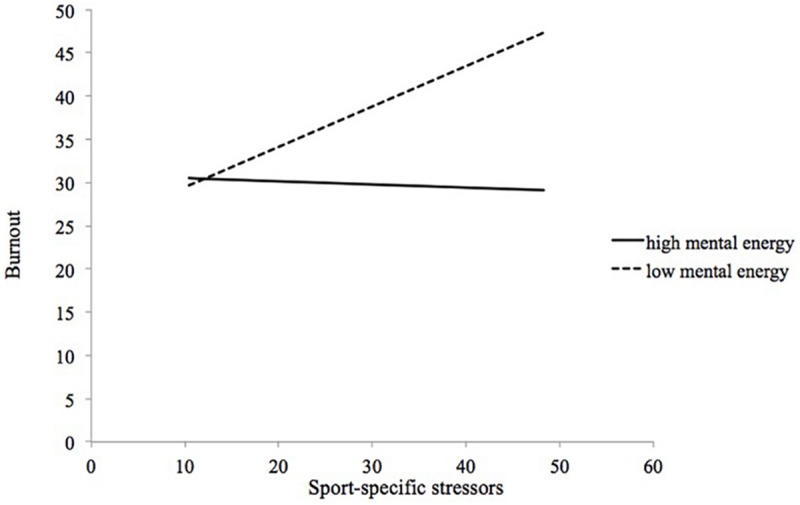
The moderating effects of total mental energy on sport-specific life stress-burnout relationship.

The interaction and simple slopes for the moderating effects of athletic mental energy on the sport-specific life stress–burnout relationship were all similar. All slopes (i.e., vigor, confidence, motivation, tireless, calm, and total mental energy score) show a slow declining pattern. To save space, we show only the figure of the moderating effect of athletic mental energy on the sport-specific life stress–burnout relationship. As Figure 1 illustrates, there is a moderating effect of athletic mental energy on the sport-specific life stress–burnout relationship, the simple slopes for low athletic mental energy is significant (*B* = 0.467, *p* < 0.01), but high athletic mental energy is not significant (*B* = −0.036, *p* = 0.77).

The simple slopes for the other five factors were similar as follows: (a) for vigor, the high vigor was *B* = 0.004 (*p* = 0.98) and the low vigor was *B* = 0.573 (*p* < 0.01); (b) for confidence, the high confidence was *B* = 0.172 (*p* = 0.18) and the low confidence was *B* = 0.518 (*p* < 0.01); (c) for motivation, the high motivation was *B* = 0.144 (*p* = 0.22) and the low motivation was *B* = 0.528 (*p* < 0.01); (d) for tireless, the high tireless was *B* = 0.064 (*p* = 0.60) and the low tireless was *B* = 0.539 (*p* < 0.01); and (e) for calm, the high calm was *B* = 0.180 (*p* = 0.15) and the low calm was *B* = 0.575 (*p* < 0.01). As earlier stated, the moderating effects of athletic mental energy on the general life stress–burnout relationship were not found in Study 1.

#### Conclusion

The purpose of Study 1 was to examine the moderating effects of athletic mental energy on the athletes’ life stress–burnout relationship. Pearson correlation analyses and hierarchical regression analyses indicated that the five factors of athletic mental energy moderated the sport-specific stress–burnout relationship. Furthermore, the interaction slopes indicated that the moderating patterns for athletic mental energy on the sport-specific life stress–burnout relationship show a slowly declining pattern. The first study provides preliminary evidence that athletic mental energy moderated the sport-specific life stress–burnout relationship.

### Study 2

#### Purpose

The purpose of Study 2 was to replicate Study 1 and provide more evidence on the moderating effects of athletic mental energy on the athletes’ stress–burnout relationship.

#### Methods

##### Participants

The participants of Study 2 were 159 college soccer players (males = 139; females = 20) with a mean age of 20.2 years (SD = ± 2.04) from 10 universities in Taiwan. On average, participants had 9.92 years (SD = ±3.38) of training and competition experience in soccer.

##### Measurements and Procedures

The data collection procedure, measurements, and statistical analyses were the same as those in Study 1. In Study 2, the Cronbach’s α of ABQ, AMES, and CASLSS were between 0.78 and 0.94 (Table 3), which show appropriate internal consistency.

**TABLE 3:** Descriptive statistics and bivariate correlations for all study variables (Study 2).

	**1**	**2**	**3**	**4**	**5**	**6**	**7**	**8**	**9**	**10**
1. SS										
2. GLS	0.61**									
3. Vigor	−0.38**	−0.29**								
4.Confidence	−0.45**	−0.38**	0.72**							
5.Motivation	−0.45**	−0.35**	0.74**	0.72**						
6.Concentration	−0.56**	−0.37**	0.63**	0.65**	0.66**					
7.Tireless	−0.40**	−0.17*	0.54**	0.54**	0.48**	0.50**				
8.Calm	−0.33**	−0.28*	0.67**	0.68**	0.62**	0.53**	0.55**			
9.Mental energy	−0.52**	−0.38**	0.87**	0.87**	0.86**	0.81**	0.72**	0.82**		
10.Brunout	0.53**	0.47**	−0.48**	−0.46**	−0.51**	−0.44**	−0.38*	−0.39**	0.54**	
Mean	2.76	2.39	4.27	4.09	4.55	4.11	3.51	4.04	4.10	2.70
SD	0.79	0.81	0.87	0.92	1.06	1.04	0.94	1.00	0.80	0.82
α	0.85	0.87	0.84	0.78	0.88	0.85	0.78	0.87	0.94	0.91

SS, sport-specific stressors; GLS, general life stressors.*p < 0.05, **p < 0.01.

#### Statistical Analyses

In Study 2, the statistical analysis procedures were similar to those of Study 1.

#### Results

As Table 3 indicates, zero-order correlations between the two types of life stress and athletic mental energy were significant (*r* = −0.17∼−0.56, *p* < 0.05). Also, the two types of life stress were positively correlated with burnout (*r* = 0.53 and 0.47, *p* < 0.01), and athletic mental energy was negatively correlated with burnout (*r* = −0.38∼−0.54, *p* < 0.01).

Table 4 shows the main predictive effects of life stress and athletic mental energy on burnout and the moderating effects of athletic mental energy on the life stress–burnout relationship. As Table 4 indicated, there are four moderating effects of athletic mental energy on the sport-specific life stress–burnout relationship: confidence (β = −0.165, Δ*R*^2^ = 0.027, *p* < 0.05), concentration (β = −0.143, Δ*R*^2^ = 0.020, *p* < 0.05), calm (β = −0.206, Δ*R*^2^ = 0.040, *p* < 0.01), and total mental energy (β = −0.159, Δ*R*^2^ = 0.025, *p* < 0.05). However, two factors of the athletic mental energy—vigor and motivation—had no significant interaction.

**TABLE 4:** Moderating effects of athletic mental energy on the sport-specific life stress-burnout relationship (Study 2).

	**Step 1: direct effect**			**Step 2: interaction effects**			
	**β**	***t*-value**	***R*^2^**	**β**	***t*-value**	***R*^2^**	**Δ*R*^2^**
SS	0.403	5.84**	0.364	0.417	6.03**	0.375	0.011
Vigor	–0.321	−4.66**		–0.318	−4.63**		
SS × Vigor				–0.105	–1.65		
SS	0.400	5.50**	0.340	–0.429	−5.93**	0.366	0.027*
Confidence	–0.282	−3.88**		–0.266	−3.71**		
SS × Confidence				–0.165	−2.55*		
SS	0.368	5.17**	0.371	0.376	5.28**	0.379	0.007
Motivation	–0.346	−4.87**		–0.328	−4.54**		
SS × Motivation				–0.088	–1.36		
SS	0.407	5.06**	0.307	0.404	5.08**	0.327	0.020*
Concentration	–0.212	−2.64**		–0.201	−2.53*		
SS × Concentration				–0.143	−2.16*		
SS	0.443	6.10**	0.311	0.437	6.05**	0.326	0.015
Tireless	–0.204	−2.81**		–0.200	−2.77**		
SS × Tireless				–0.122	–1.85		
SS	0.445	6.39**	0.328	0.491	7.09**	0.368	0.040**
Calm	–0.242	−3.48**		–0.236	−3.48**		
SS × Calm				–0.206	−3.15**		
SS	0.335	4.50**	0.372	0.356	4.83**	0.397	0.025*
Mental energy	–0.363	−4.88**		–0.343	−4.66**		
SS × Mental energy				–0.159	−2.53**		

SS, sport-specific stressors; Mental energy, total mental energy score.*p < 0.05, **p < 0.01.

**FIGURE 2:**
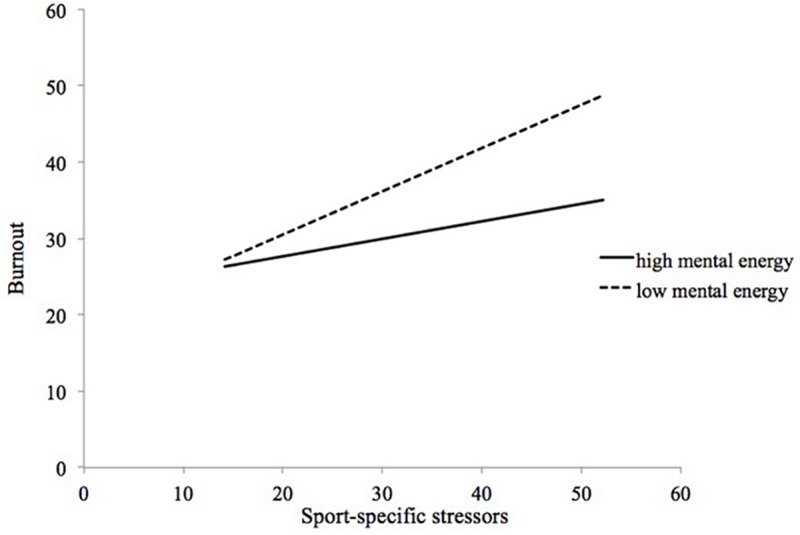
Moderating effect of total athletic mental energy on sport-specific life stress-burnout relationship.

Similar to Study 1, the interaction and simple slopes of the moderating effects of athletic mental energy on the sport-specific life stress–burnout relationship are similar. All figures (i.e., confidence, concentration, calm, and total mental energy score) show a slowly declining pattern. Again, we present only the figure of the moderating effect of the total athletic mental energy on the sport-specific life stress–burnout relationship. As Figure 2 illustrates, the simple slopes indicate that there is a moderating effect of athletic mental energy on the sport-specific life stress–burnout relationship. The simple slopes for both high (*B* = 0.310, *p* < 0.01) and low (*B* = 0.681, *p* < 0.01) athletic mental energy score are all significant. The simple slopes for the other four factors were similar: (a) for concentration, the high concentration was *B* = 0.298 (*p* < 0.05) and the low concentration was *B* = 0.604 (*p* < 0.01); (b) for confidence, the high confidence was *B* = 0.308 (*p* < 0.01) and the low confidence was *B* = 0.650 (*p* < 0.01); and (c) for calm, the high calm was *B* = 0.330 (*p* < 0.01) and the low calm was *B* = 0.767 (*p* < 0.01).

Unlike Study 1, in Study 2, we found both main predictive effects and moderating effects of the athletic mental energy on the general life stress–burnout relationship. Table 5 shows the main predictive effects of general life stress and athletic mental energy on burnout and the moderating effects of athletic mental energy on the general life stress–burnout relationship. As step 2 indicated, there are three moderating effects of athletic mental energy on the general life stress–burnout relationship: concentration (β = −0.191, Δ*R*^2^ = 0.036, *p* < 0.01), tireless (β = −0.210, Δ*R*^2^ = 0.042, *p* < 0.01), and total mental energy (β = −0.134, Δ*R*^2^ = 0.017, *p* < 0.05).

**TABLE 5:** Moderating effects of athletic mental energy on general life stress-burnout relationship (Study 2).

	**Step 1: direct effect**			**Step 2: interaction effects**			
	**β**	***t*-value**	***R*^2^**	**β**	***t*-value**	***R*^2^**	**Δ*R*^2^**
GLS	0.368	5.47**	0.350	0.388	5.58**	0.355	0.005
Vigor	–0.369	−5.48**		–0.361	−5.34**		
GLS × Vigor				–0.076	–1.14		
GLS	0.349	4.87**	0.316	0.366	−5.03**	0.323	0.007
Confidence	–0.326	−4.55**		–0.319	−4.44**		
GLS × Confidence				–0.086	–1.27		
GLS	0.334	4.88**	0.361	0.335	4.86**	0.361	0.000
Motivation	–0.395	−5.77**		–0.393	−5.41**		
GLS × Motivation				–0.004	–0.65		
GLS	0.360	5.00**	0.304	0.356	5.06*	0.341	0.036**
Concentration	–0.305	−4.23**		–0.290	−4.12**		
GLS × Concentration				–0.191	−2.92**		
GLS	0.422	6.29**	0.319	0.434	6.66**	0.361	0.042**
Tireless	–0.311	−4.65**		–0.349	−5.27**		
GLS × Tireless				–0.210	−3.20**		
GLS	0.396	5.68**	0.297	0.433	5.86**	0.307	0.010
Calm	–0.280	−4.02**		–0.276	−3.97**		
GLS × Calm				–0.104	–1.47		
GLS	0.317	4.64**	0.376	0.343	4.99**	0.394	0.017*
Mental energy	–0.420	−6.16**		–0.402	−5.91**		
GLS × Mental energy				–0.134	−2.10**		

GLS, general life stressors; Mental energy, total mental energy score. *p < 0.05, **p < 0.01.

**FIGURE 3:**
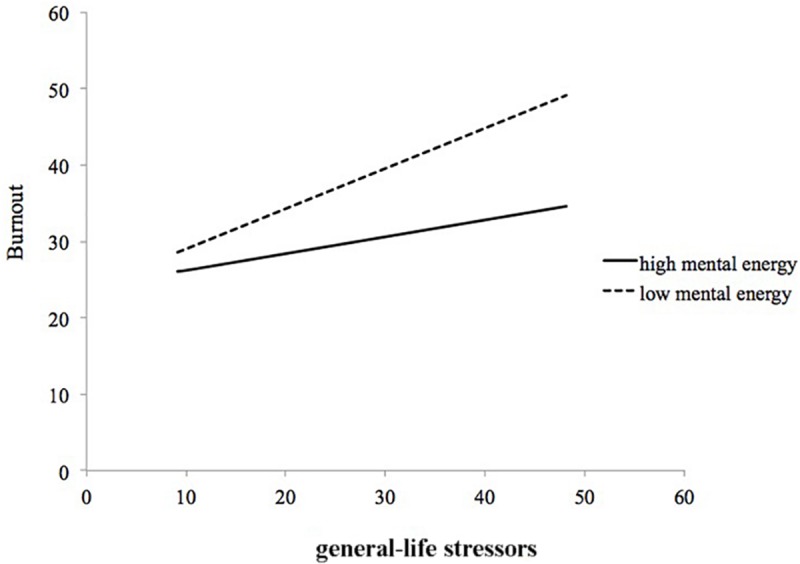
Moderating effect of total athletic mental energy on the general- life stress-burnout relationship.

The figures of the moderating effects of athletic mental energy on the general life stress–burnout relationship are also similar. All figures (i.e., concentration, tireless, and total mental energy score) show a slowly declining pattern. To save space, we only show the figure of the moderating effect of the total athletic mental energy on the general life stress–burnout relationship. As Figure 3 illustrates, the simple slopes indicate that there is a moderating effect of athletic mental energy on the general life stress–burnout relationship. The slopes for both high (*B* = 0.220, *p* < 0.05) and low (*B* = 0.527, *p* < 0.01) athletic mental energy are all significant. The simple slopes for the other three factors were similar: (a) for concentration, the simple slope for high was *B* = 0.195 (*p* > 0.05) and the low was *B* = 0.581 (*p* < 0.01); (b) for tireless, the high was *B* = 0.251 (*p* < 0.05) and the low was *B* = 0.697 (*p* < 0.01); and (c) for total athletic mental energy, the high was *B* = 0.220 (*p* < 0.05) and the low was *B* = 0.527 (*p* < 0.01).

#### Conclusion

The purpose of Study 2 was to replicate Study 1 to examine the moderating effects of athletic mental energy on the athletes’ life stress–burnout relationship. With a different sample, we found that four factors of athletic mental energy moderated the sport-specific stress–burnout relationship, and three factors of athletic mental energy moderated the general life stress–burnout relationship. Furthermore, all of the interaction slopes indicated that the moderating patterns for athletic mental energy on two types of the life stress–burnout relationship show a slowly declining pattern. Thus, Study 2 replicated the results from Study 1, but with added evidence. The theoretical implications, strengths, and limitations of the study and applications and future direction for the research are discussed in the following section.

## Discussion

### Theoretical Contributions/Implications

In search of positive strengths that moderate the athletes’ stress–burnout relationship, we extended Lu et al. (2018) work on athletic mental energy and examined its moderating effects on the athletes’ stress–burnout relationship. Across two studies, we consistently found that athletic mental energy moderated the athletes’ life stress–burnout relationship. Study 1 found that five factors of athletic mental energy (i.e., vigor, confidence, motivation, tireless, and calm) moderated the athletes’ sport-specific life stress–burnout relationship but not the general life stress–burnout relationship. Study 2 found that three factors of athletic mental energy (i.e., confidence, concentration, and calm) moderated the athletes’ sport-specific life stress–burnout relationship. Also, in Study 2, we found that two factors of athletic mental energy (i.e., concentration and tireless) moderated the general life stress–burnout relationship. Thus, our study provides initial evidence that athletic mental energy can be a positive strength in protecting athletes’ psychological well-being from stress-induced burnout. This is the major contribution of the study. Also, our study provides several theoretical implications for the researchers.

First, like other positive strengths in sport studies, athletic mental energy negatively correlated with burnout. Specifically, the moderating effects of athletic factors on the stress–burnout relationship were consistent over Study 1 and Study 2. Worthy to note is that three emotional components of athletic mental energy (i.e., vigor, tireless, and calm) played important roles in moderation. Vigor is an individual’s subjective feeling with heightened arousal. Along with heightening vigor, an individual would maximize his/her efforts in enhancing performance (Lane and Terry, 2000). Research indicates that when individuals encounter adversities/challenges in life, if they can exert more effort to address the problems and overcome obstacles, they can get back to homeostasis physically, socially, and psychologically (Lazarus and Folkman, 1984). Thus, it may be that those athletes with high vigor exert more effort to cope with stressors in sports, and thus they did not experience burnout as much as their counterparts did. Similar explanations can be applied to the moderating effects of tireless on the sport-specific life stress–burnout relationship. In Lu et al. (2018) study, tireless was derived from vigor through factor analyses.

The moderating effects of calm in sport-specific life stress are very insightful. Past research in elite sports found that athletes in peak performance experienced a state of calm such as “no fear of failure” and “physically and mentally relaxed” (Lohr, 1984, p. 67) even when competition environments are very stressful. In medical care settings, it is found that nurses in the intensive care department are very stressed—sometimes they need 24 h or 7 days of work to treat an emergency patient. Facing a stressful working condition, it is found that those nurses remaining calm and confident can adapt and accomplish the mission (Ennis et al., 2014). Therefore, it is possible that in a stressful environment, such as sports training and competition, those athletes with the positive emotion of calm might cope well so they do not experience burnout.

There are several positive cognitive components of athletic mental energy in moderating the athletes’ life stress–burnout relationship that need further discussion. Specifically, both in Study 1 and Study 2, confidence moderated the sport-specific life stress–burnout relationship. It has been found that athletes high in confidence has lower pre-competition anxiety and performed better (Nicholls et al., 2010). According to Vealey and Chase’s (2008) sport–confidence model, high confidence may trigger positive emotions and greater effort to deal with adversities in sports. Thus, the positive element of confidence in athletic mental energy may help athletes cope with sport-specific life stressors (e.g., performance demands, sports injury, training adaptation, and coach–athlete relationship) because they can exert more effort to overcome difficulties.

The other cognitive element of athletic mental energy in moderating the sport-specific life stress–burnout relationship is concentration. Concentration refers to one’s cognitive ability to block distractions and focus one’s attention to a given task (Weinberg and Gould, 2015). Research has found that athletes who perform better in important games scored high in concentration (Abdullah et al., 2016). Also, research investigating athletes’ mental state in the peak performance indicated that “able to focus tasks at hand” and “emerge in the activity that they engage” are major characteristics at this moment (Williams et al., 2013). In contrast, research also found that if an individual cannot concentrate on work during threatening or demanding situations, he/she might have reduced ability to focus, impaired information processing, and decreased working memory (Gaillard, 2018). Thus, athletes low in athletic mental energy, particularly concentration, would be unable to focus their attention on given tasks, such as competition or training. Consequently, they would not be able to handle those sport-specific demands, which in turn, increase stress. Over the long term, those low in concentration would be high in stress and stress-induced burnout.

The moderating effect of motivation on the sport-specific life stress–burnout relationship in Study 1 is unique. Motivation refers to the intensity and direction of behavior and why people behave as they do (Gill and Williams, 2008). Generally, highly motivated individuals tend to persist and strive in their goal-directed behavior. Research has found that high achievers in sports increase efforts and persist in the pursuit of their goals when encountering adversities, such as injury and failure (Sarkar et al., 2015). Furthermore, it was found that those athletes who engage in sports purely for the intrinsic reasons such as fun and enjoyment are working hard during seasons, which subsequently predicted their end-season goal attainments (Smith et al., 2011). Thus, those athletes with the high motivation of athletic mental energy would be able to exert more effort to handle the demands in sports either in training, preventing injury, or maintaining a good coach–athlete relationship. By doing so, they would not experience those stressors derived from sports participation.

There are several differences between the two studies. For example, Study 1 found more moderators that buffered the sport-specific life stress–burnout relationship than Study 2. In contrast, Study 2 found that two factors of athletic mental energy (i.e., concentration and tireless) moderated the general life stress–burnout relationship. The reasons for these differences are complicated because the participants in Study 2 were soccer players. Soccer is a team sport that requires teamwork and cooperation between teammates to achieve the team’s goal. Whether the nature of sport causes the differences in our study needs further examination. Study 2’s finding that athletic mental energy also moderated the general life stress–burnout relationship can be explained by the transfer effects of life skills in sports to general living conditions. Research suggests that athletes may learn behavioral, cognitive, interpersonal, and intrapersonal skills from sports and transfer to their daily lives (Gould and Carson, 2008). Thus, even in general life condition, athletes’ high athletic mental energy is beneficial to handle daily life stressors. However, this is only one possibility. Future studies may examine how athletic mental energy helps athletes handle their adversities in daily life.

### Practical Applications

Athletic mental energy is a newly emerging topic in sports and psychology. Research has found that athletic mental energy predicts winning and losing in martial arts (Lu et al., 2018), negatively correlates with life stress and burnout (Lu et al., 2018; and this study), positively correlates with athletes’ positive state of mind (Lu et al., 2018), and moderated the athletes’ stress–burnout relationship in this study. Thus, sports coaches, sport psychologists, athletes, and sports professionals can apply athletic mental energy in their professional practices. According to Lu et al. (2018), athletic mental energy is influenced by many personal and environmental factors, such as life patterns, nutrition, sleep, interpersonal relationship, and time management, and can be gained from mental and physical training. Therefore, coaches and sport psychologists can schedule psychological skills training (PST) in athletes’ daily training to increase athletic mental energy. Also, because athletic mental energy might vary with nutrition or life management, athletes need a healthy diet and a regular life schedule to sleep well at night.

### Limitations and Future Suggestions

There are several limitations in our research. First, although we found moderating effects of athletic mental energy on the athletes’ stress–burnout relationship, due to the cross-sectional nature, the results do not imply a causal relationship. We suggest that future studies adopt a longitudinal design to investigate athletes’ life stress, athletic mental energy, and burnout over time to examine causal effects. Second, the participants in this study were all student-athletes. Therefore, whether the results can be generalized to other athletes such as professional athletes or junior athletes needs to be further examined. Furthermore, the data were collected from Taiwanese student-athletes; whether the results can be generalizable to different cultures needs to be further studied in the future. Moreover, we just sampled soccer players in Study 2; we suggest that future studies may sample individual sports athletes, such as track and field, gymnastics, and swimming, to examine the possible moderating effects of athletic mental energy on the stress–burnout relationship.

## Conclusion

In search of positive strengths that may moderate the athletes’ stress–burnout relationship, we conducted two studies to examine the moderating effects of athletic mental energy on the athletes’ stress–burnout relationship. Results consistently found that athletic mental energy can be positive strengths for athletes in buffering their life stress and stress-induced burnout. We hope that more research will explore the positive effects of athletic mental energy in the sports domain not only for the enhancement of the performance but also for the promotion of the athletes’ psychological well-being.

## Data Availability Statement

The datasets analyzed in this manuscript are not publicly available. Requests to access the datasets should be directed to frankjlu@gmail.com.

## Ethics Statement

The studies involving human participants were reviewed and approved by the Antai Medical Care Cooperation Antai-Tian-Sheng memorial Hospital Institutional Review Board. The patients/participants provided their written informed consent to participate in this study.

## Author Contributions

All authors listed have made a substantial, direct and intellectual contribution to the work, and approved it for publication.

## Conflict of Interest

The authors declare that the research was conducted in the absence of any commercial or financial relationships that could be construed as a potential conflict of interest.

## Figures and Tables

**TABLE 6:** Athletic Mental Energy Scale (AMES).

**Directions:** Below are 18 statements that describe **“how do you feel right now”** in sports		**Completely**	**Hardly**	**A little**	**Much**	**Very**	**Completely**
training/competition. Please circle a number that mostly represents your feeling.		**not**	**ever**	**bit**		**much**	**so**
**1**	**I feel spiritual to do everything in sports**	**1**	**2**	**3**	**4**	**5**	**6**
2	I feel there is endless energy coming from my body	1	2	3	4	5	6
**3**	**I feel I can win all the competitions in the future**	**1**	**2**	**3**	**4**	**5**	**6**
4	I feel excited in future competitions	1	2	3	4	5	6
**5**	**There’s nothing distracting me in competition**	**1**	**2**	**3**	**4**	**5**	**6**
6	There’s nothing distracting me in training	1	2	3	4	5	6
**7**	**No matter how long the training lasts I don’t feel tired**	**1**	**2**	**3**	**4**	**5**	**6**
8	I am full of passion to attend my sports	1	2	3	4	5	6
**9**	**I can have my sports movements and skills automatically executed in sports**	**1**	**2**	**3**	**4**	**5**	**6**
10	I am free of distraction during competition and training	1	2	3	4	5	6
**11**	**Even the competition is over I still feel I have endless energy to use**	**1**	**2**	**3**	**4**	**5**	**6**
12	Even the training is over I still feel I have endless energy to use	1	2	3	4	5	6
**13**	**I can control all sports movements and skills**	**1**	**2**	**3**	**4**	**5**	**6**
14	When facing to my opponents I am calm	1	2	3	4	5	6
**15**	**Either in competition or training, I feel full of energy**	**1**	**2**	**3**	**4**	**5**	**6**
16	I want to show my best to others in sports	1	2	3	4	5	6
**17**	**Facing coming competitions I don’t feel anxious**	**1**	**2**	**3**	**4**	**5**	**6**
18	Even facing a tough opponent I don’t feel anxious	1	2	3	4	5	6

*#1, AMES is scored as follows: (a) vigor = 1, 2, 15; (b) confidence = 3, 9, 13; (c) motivation = 4, 8, 16; (d) concentration = 5, 6, 10; (e) tireless = 7, 11, 12; and (f) calm = 14, 17, 18. #2, total AMES is scored by summing all subscales.*
